# Splenectomy during cytoreductive surgery in advanced epithelial ovarian cancer can be predicted

**DOI:** 10.1016/j.eurox.2025.100395

**Published:** 2025-05-09

**Authors:** Thien-Kim Do, Yohann Dabi, Cyril Touboul, Jennifer Uzan, François Margueuritte, Geoffroy Canlorbe, Yohan Kerbage, Vincent Lavoué, Chérif Akladios, Lobna Ouldamer, Hélène Costaz, Alexandre Bricou, Henri Azaïs, Pauline Chauvet, Xavier Carcopino, Cyrille Huchon, Camille Mimoun

**Affiliations:** a Sorbonne University - Department of Gynaecology and Obstetrics, Hôpital Tenon, Assistance Publique-Hôpitaux de Paris (AP-HP), Paris 75020, France; b Department of Obstetrics Gynecology and Reproductive Medicine, University Paris Est Créteil, Centre Hospitalier Inter-Communal de Créteil, Creteil 94000, France; c Department of Gynaecology, Centre Hospitalier Intercommunal de Poissy-Saint-Germain-en-Laye Site Hospitalier de Poissy, Poissy 78300, France; d Department of Gynecological and Breast Surgery and Oncology, Pitié-Salpêtrière, AP-HP, University Hospital, Paris 75013, France; e Department of Gynecologic Surgery, Jeanne de Flandre Hospital, CHU de Lille, Avenue Eugène Avinée, Loos 59120, France; f Department of Gynaecological Surgery, INSERM U1085, équipe 8, CRLC Eugène Marquis, Université de Rennes 1, Hôpital Sud, CHU de Rennes, Rennes 35000, France; g Department of Gynaecology, Hôpitaux Universitaires de Strasbourg, Strasbourg 67000, France; h Department of Gynaecology, Hôpital Universitaire de Tours, Tours 37000, France; i Department of Surgical Oncology, Georges, Francois Leclerc Centre, Dijon 21231, France; j Department of Gynecology, Bobigny University, AP-HP, Jean-Verdier Hospital, Bondy 93140, France; k Gynecologic and Breast Oncologic Surgery Department, Georges Pompidou European Hospital, APHP Centre, Paris 75015, France; l Department of Gynaecology, CHU de Clermont Ferrand, Clermont Ferrand 63003, France; m Department of Obstetrics and Gynaecology, Hôpital Nord, APHM, Aix-Marseille University (AMU), University Avignon, CNRS, IRD, IMBE, UMR 7263, Marseille 13005, France; n Department of Gynecology and Obstetrics, Lariboisiere Hospital, AP-HP, University of Paris Cité, Paris 75010, France

**Keywords:** Splenectomy, Advanced epithelial ovarian cancer, Cytoreductive surgery, Predictive factors, Score, Risk group

## Abstract

**Introduction:**

Splenectomy may be necessary for complete cytoreductive surgery (CRS) in advanced stage epithelial ovarian cancer (AS-EOC), potentially raising perioperative morbidity and necessitating specific patient management.

**Objective:**

This study aimed to develop a predictive score of splenectomy in CRS of AS-EOC.

**Materials and methods:**

Data from histologically confirmed AS-EOC (FIGO IIB-IV) before CRS and diagnosed between 01/01/2000 and 01/06/2017, were extracted from the FRANCOGYN multicentric database (14 French hospitals). After identifying predictive factors of splenectomy, we performed a logistic regression to develop a prediction model and construct a risk score, allowing identification of a high-risk group. Model discrimination was assessed using a Receiver Operating Characteristic (ROC) curve. Decision Curve Analysis (DCA) was then conducted to evaluate the model’s net clinical benefit across a range of threshold probabilities.

**Results:**

Among 1288 patients included, 7 % (n = 91) underwent splenectomy. Four independent variables statistically associated with splenectomy were identified: age < 60 years (aDOR = 1.76, 95 % CI [1.13–2.75], p = 0.015), omental cake (aDOR = 2.12, 95 % CI [1.11–4.08], p = 0.024), diaphragmatic carcinosis (aDOR = 2.36, 95 % CI [1.34–4.18], p = 0.001), and digestive involvement at initial CT and/or laparoscopy (aDOR = 3.24, 95 % CI [1.93–5.43], p < 0.001). The ROC-AUC of this prediction model was 0.76. Patients meeting all 4 criteria with a maximum of 10 points defined the high-risk group and had a splenectomy probability of 32 % (95 % CI [22.00–44.31]), with a specificity of 95.8 % (95 % CI [94.5–96.9]) and a positive likelihood ratio of 6.31 (95 % CI [4.08–9.78]). The DCA showed a positive net clinical benefit of the model between 15 % and 40 % threshold probabilities.

**Conclusion:**

Using a simple 4 – variable predictive score, patients at high risk of splenectomy during CRS in AS-EOC could be identified to improve patients’ preoperative information and perioperative management.

## 1 Introduction

Ovarian cancer is the eighth most common cancer among women in France and second deadliest. In 2018, 5 193 ovarian cancers were diagnosed and mortality rate was estimated to 3 479 [1]. Generally diagnosed at an advanced stage (stage IIB-IV) according to the 2018 classification of the International Federation of Gynaecology and Obstetrics (FIGO), prognosis remains relatively poor with a 5-year survival rate around 40 % [2], [3].

Standard treatment of advanced epithelial ovarian cancer (AEOC) relies on cytoreductive surgery (CRS) and chemotherapy based on taxanes and platinum [4], [5]. Complete CRS with no residual macroscopic disease is the major prognosis factor [6], [7]. Thus, optimal preoperative evaluation of resectability by preoperative imaging and diagnostic laparoscopy is essential [8]. In patients with extensive carcinomatosis not resectable in primary CRS, interval/final CRS after neoadjuvant chemotherapy (NACT) can be proposed [9], [10]. Extensive CRS including multiple organ resections such as splenectomy, resection of diaphragm dome or hepatectomy can be performed if required.

In the literature, splenectomy rate varies between 1.3 % and 20 %. Splenectomy can be indicated in 3 situations: parenchymal disease, presence of carcinomatosis lesions on the capsule or on the splenic hilum and to resolve a visceral or vascular injury [11]. Splenic metastases are associated with more abdominal spread carcinomatosis and thus more extensive CRS including multiple intestinal resections or partial hepatectomy [12], [13], [14]. Even if most studies reported splenectomy to be safe and feasible with no impact on survival [15], [16], [17], [18], its frequent association with those other surgical procedures could increase patient morbidity (about 15–30 %) [19], [20] and sometimes even justify to postpone the primary CRS for NACT.

Therefore, the prediction of splenectomy could be a valuable information for the global management of patients. As an example, its anticipation can allow patient education for vaccination and ensuring the presence of a visceral surgeon during the procedure. Only two studies mention preoperative evaluation of spleen extension and concordance with imaging. According to Uzan et al. and Davies et al. [18], [20] the prediction of splenectomy before surgery is around 20 % by imaging (only CT). So far, no predictive factors of splenectomy have been identified.

The aim of this multicentric and retrospective study is to develop a predictive score of splenectomy in primary or interval/final CRS for AEOC.

## 2 Materials and methods

### 2.1 Population and data

We conducted a retrospective, multicentric cohort study in fourteen referral centers for ovarian cancer in France within the FRANCOGYN study group (Lariboisiere University Hospital, Tenon University Hospital, Intercommunal Hospital of Creteil, Jean Verdier University Hospital, La Pitie Salpêtrière University Hospital, Poissy University Hospital, European George Pompidou University Hospital, Lille University Hospital, Tours University Hospital, Rennes University Hospital, Georges-Francois Leclerc Dijon Hospital, Strasbourg University Hospital, Marseille University Hospital and Clermont Ferrand University Hospital).

Patients with histologically confirmed AEOC, FIGO IIB-IV prior to CRS, and diagnosed between 01/01/2000 and 01/06/2017 were included. Epithelial ovarian cancer included serous, mucinous, endometrioid, clear cell and undifferentiated subtypes. The following patients were not included: (i) patients with non-epithelial ovarian cancer and/or early stage and/or not determined ovarian cancer; (ii) patients never operated on, either because of non resectable disease despite neoadjuvant chemotherapy or poor physical condition.

The research protocol was approved by the Ethics Committee for Research in Obstetrics and Gynecology (CEROG 2019-GYN-604).

The following clinical and demographic variables were abstracted. The data were fully anonymized before we accessed them. The data were age at diagnosis, body mass index (BMI), gestity, parity, menopausal status, personal comorbidities, American Society of Anesthesiologists (ASA) score and presence or absence of identified genetic mutations. Preoperative assessment was reported with imaging (CT and PET-CT), diagnostic laparoscopy with notably Peritoneal Carcinomatosis Index score according to Sugarbaker (PCI) [21] and FIGO stage including lymph node staging [2]. Tumor characteristics were noted including histological type and tumor grade. Preoperative CA 125 level was also collected. Then, data on treatment were recorded. If neoadjuvant chemotherapy was indicated, number of cycles and therapeutic agents were assessed.

### 2.2 Surgical technique

Timing of CRS (primary or interval/final) and each surgical procedures during laparotomy were precisely noted. All patients underwent median laparotomy.

After a thorough exploration, if the patient was confirmed resectable, CRS with at least total hysterectomy, bilateral salpingo-oophorectomy, infra gastric omentectomy and the removal of any other intraperitoneal metastasis can be completed. Pelvic and para aortic lymphadenectomy were performed according to guidelines at the time of surgery but could be omitted depending on preoperative imaging and patient tolerance of the surgical procedure [4], [22]. The completeness of CRS could require douglassectomy, digestive resection and/or diaphragmatic resection.

If splenic parenchymal metastasis was expected, if the spleen was covered with lesions or if the omental cake infiltrates the splenic capsule or hilum, splenectomy was performed. To complete splenectomy, the splenic flexure of colon was mobilized by coagulating the splenocolic ligament and cutting through the fascia of Toldt. All the short gastric vessels and the splenophrenic ligament were coagulated and cut. Then, a dissection of the splenic hilum and pancreas tail allowed to electively ligate and section the splenic artery and vein. If the purpose of splenectomy was to resolve spleen bleeding, the splenic hilar vessels were first treated.

Residual tumor at the end of intervention was assessed and defined by complete resection (CC0) when there were no macroscopic residual disease; CC1, residual tumor of 1–10 mm; CC2, residual tumor >10 mm [23], [24].

### 2.3 Statistical analysis

Statistical analysis were performed using STATA 13.0 (Stata Corp.; College Station, TX, USA). p values <0.05 were considered significantly different.

We compared two groups: “No splenectomy” and “Splenectomy” groups.

Univariable analysis using a quantitative (Student's *t*-test) or a qualitative (Chi2 test) test was performed. Some quantitative variables were dichotomized to maximize their accuracy value. The accuracy of each predictive factor of splenectomy was assessed based on sensitivity (Se), specificity (Sp), positive likelihood ratio (LR+), negative likelihood ratio (LR-) and diagnostic odds ratio (DOR).

The multiple logistic regression analysis was used to select the best combination of variables that was independently associated with splenectomy (p < 0.05). Variables were selected by a backward stepwise procedure from those associated with splenectomy in the univariate analysis at a threshold of p < 0.20. Adjusted diagnostic odds ratios (aDOR) were calculated. The stability of each variable in the model was tested using a bootstrap procedure of 1000 repetitions [25]. The discrimination of the prediction logistic regression model was specified by calculating its area under the ROC curve (ROC-AUC) [26].

A splenectomy predictive score by rounding up the β coefficients from the logistic regression model to generate a simple scale was built [27]. The ROC-AUCs of the model and of the splenectomy predictive score were compared to check that the two values were not significantly different. Missing data were considered as a distinct category in the logistic regression model to avoid patient exclusion from the multivariable analysis. We created a high-risk group of splenectomy by choosing the threshold value of the splenectomy predictive score with a specificity >90 % and a LR+> 4 (rule in) [27].

A Decision Curve Analysis (DCA) was conducted to evaluate the clinical utility of the predictive score. The analysis compared the net benefit of using the model across a range of threshold probabilities (from 1 % to 50 %) against two reference strategies: treating all patients as candidates for splenectomy and treating none. Predicted probabilities were derived by normalizing the individual scores (0–10 points) to a 0–1 scale. Net benefit was calculated as the difference between true positives and the weighted proportion of false positives, providing insight into the range where the model could offer superior clinical value in guiding perioperative decision-making.

## 3 Results

### 3.1 Population

Between January 2000 and June 2017, 2 712 patients were operated for an ovarian cancer within one of the participating centers. Of these, 1 288 patients fitted inclusion criteria: n= 91 patients (7 %) underwent a splenectomy during CRS and n= 1 197 patients (93 %) did not.

Main characteristics of the patients included are presented in Table 1. The two groups did not differ statistically except for age (p = 0.02), FIGO stage (p < 0.003) and initial CA125 (p < 0.001).

**Table 1:** Clinical, tumor, biological and surgical characteristics of the population.

**Variables**	**All Population** **N = 1288 (%)**	**No Splenectomy** **n = 1197 (%)**	**Splenectomy** **n = 91 (%)**	**p**
**CLINICAL CHARACTERISTICS**				
**Age (years) (mean +/- SD)**	62.02 +/- 11.43	62.21 +/- 11.42	59.42 +/- 11.18	0.02
**BMI (kg/m**^**2**^**) (mean +/- SD)**	62.63 +/- 141.29	60.36 +/- 137.87	99.53 +/- 186.16	0.12
**Gestity (mean +/- SD)**	2.03 +/- 1.67	2.02 +/- 1.69	2.14 +/- 1.51	0.52
**Parity (mean +/- SD)**	1.80 +/- 1.26	1.80 +/- 1.47	1.82 +/- 1.26	0.93
**Menopause**	1087 (86.68)	1009 (86.76)	78 (85.71)	0.78
**Mutation**				0.57
- **No mutation**	395 (70.16)	364 (69.60)	31 (77.50)	
- **BRCA/BRCA2**	147 (26.11)	139 (26.58)	8 (20.00)	
- **Others**	21 (3.73)	20 (3.82)	2 (2.50)	
**ASA score**				0.44
- **1** - **2** - **3** - **4** - **5**	360 (38.14) 454 (48.09) 124 (13.14) 5 (0.53) 1 (0.11)	324 (37.28) 423 (46.68) 116 (13.35) 5 (0.58) 1 (0.12)	36 (48.00) 31 (41.33) 8 (10.67) 0 (0.00) 0 (0.00)	
**Medical or surgical history**				0.87
- **No** - **Yes**	306 (34.23) 588 (65.77)	279 (34.15) 538 (65.85)	27 (35.06) 50 (64.94)	
**TUMOR CHARACTERISTICS**				
**Initial FIGO stage**				0.003
- **2** - **3** - **4**	70 (5.43) 986 (76.55) 232 (18.01)	69 (5.76) 923 (77.11) 205 (17.13)	1 (1.10) 63 (69.23) 27 (29.67)	
**Tumor histological type**				0.15
- **Serous** - **Endometrioid** - **Mucinous** - **Clear-cell carcinoma** - **Transitional-cell carcinoma**	1142 (88.66) 73 (5.67) 21 (1.63) 49 (3.80) 3 (0.23)	1061 (88.64) 68 (5.68) 18 (1.50) 48 (4.01) 2 (0.17)	81 (89.01) 5 (5.49) 3 (3.30) 1 (1.10) 1 (1.10)	
**BIOLOGICAL CHARACTERISTICS**				
**Initial CA125 level (IU/L) (mean +/- SD)**	1687.32 +/- 4669.87	1649.77 +/- 4721.944	2172.73 +/- 3927.58	0.26
**SURGICAL CHARACTERISTICS**				
**Initial PCI score (mean +/- SD)**	12.18 +/- 9.44	11.69 +/- 9.38	17.27 +/- 8.63	<0.001
**Time of CRS**				0.65
- **Primary** - **Interval/Final**	793 (61.57) 495 (38.43)	739 (61.74) 458 (38.26)	54 (59.34) 37 (40.66)	

SD: Standard Derivation; BMI: Body Mass Index; BRCA: BReast CAncer; ASA: American Society of Anaesthesiologists; FIGO: Fédération Internationale de Gynécologie-Obstétrique; CA: Carcinome Antigen; PCI: Peritoneal Carcinomatosis Index.

### 3.2 Univariate analysis

Univariate analysis for predictive factors of splenectomy is presented in Table 2.

**Table 2:** Univariate analysis for predictive factors of splenectomy.

**Variables**	**Total, n/N**	**No Splenectomy,** **n (%)**	**Splenectomy,** **n (%)**	**Se (%)**	**Sp (%)**	**LR+**	**LR-**	**DOR** **95 % CI**	**p**
**CLINICAL CHARACTERISTICS**									
**Age <60 years**	517/1287	472 (39.46)	45 (49.45)	49.5	60.5	1.25	0.84	1.50 0.98–2.29	0.06
**Menopause**	1087/1254	1009 (86.76 %)	78 (86.71)	85.7	13.2	0.99	1.08	0.92 0.50–1.67	0.78
**BRCA/other mutation**	168/563	159 (30.40)	9 (22.50)	22.5	69.6	0.74	1.11	0.66 0.31–1.41	0.29
**TUMOR CHARACTERISTICS**									
**Serous histological type**	1142/1288	1061 (88.64)	81 (89.01)	89.0	11.4	1.00	0.97	1.04 0.53–2.03	0.91
**Mucinous histological type**	21/1288	18 (1.50)	3 (3.30)	3.3	98.5	2.19	0.98	2.23 0.69–7.25	0.19
**INITIAL RADIOLOGICAL CHARACTERISTICS: CT**									
**One ovary involvement**	545/944	518 (59.00)	27 (40.91)	40.9	41.0	0.69	1.44	0.48 0.39–0.80	0.004
**Two ovaries involvement**	302/713	271 (41.44)	31 (52.54)	52.5	58.6	1.27	0.81	1.56 0.92–2.67	0.10
**Carcinomatosis**	420/566	385 (72.64)	35 (97.22)	97.2	27.4	1.34	0.10	13.18 1.75–99.14	0.001
**Diaphragmatic carcinosis**	165/904	142 (16.92)	23 (35.38)	35.4	83.1	2.09	0.78	2.69 1.56–4.63	0.000
**Omentum carcinosis**	395/921	364 (42.42)	31 (49.21)	49.2	57.6	1.16	0.88	1.31 0.79–2.20	0.29
**Small intestine involvement**	50/835	47 (6.06)	3 (5.08)	5.1	93.9	0.84	1.01	0.83 0.25–2.76	0.76
**Colon involvement**	106/903	91 (10.87)	15 (22.73)	22.7	89.1	2.09	0.97	2.41 1.30–4.48	0.004
**Liver metastasis**	64/1018	55 (5.80)	9 (13.04)	13.0	94.2	2.25	0.92	2.44 1.15–5.18	0.02
**Ascitis**	535/908	486 (57.86)	49 (72.06)	72.1	42.1	1.25	0.66	1.88 1.08–3.25	0.02
**Pelvic LNM**	209/908	196 (23.14)	13 (21.31)	21.3	76.9	0.92	1.02	0.90 0.48–1.70	0.74
**Para-aortic LNM**	204/840	189 (24.26)	15 (24.59)	24.6	75.7	1.01	1.00	1.02 0.56–1.87	0.95
**Supra-diaphragmatic LNM**	100/828	93 (12.27)	7 (10.00)	10.0	87.7	0.82	1.03	0.79 0.35–1.79	0.58
**Pleural effusion**	160/1058	147 (14.97)	13 (17.11)	17.1	85.0	1.14	0.97	1.17 0.63–2.18	0.62
**INITIAL BIOLOGICAL CHARACTERISTICS**									
**CA125 level ≥500**	620/1142	568 (53.58)	52 (63.41)	63.4	46.4	1.18	0.79	1.50 0.96–2.38	0.09
**INITIAL LAPAROSCOPY CHARACTERISTICS**									
**PCI score ≥15**	272/714	236 (36.10)	37 (58.73)	58.7	63.9	1.63	0.65	2.52 1.49–4.25	0.000
**Omental cake**	402/684	343 (55.59)	59 (88.06)	88.1	44.4	1.58	0.27	5.89 2.81–12.34	0.000
**Peritoneal carcinosis**	498/807	430 (58.74)	68 (90.67)	90.7	41.3	1.54	0.23	6.82 3.15–14.78	0.000
**Diaphragmatic carcinosis**	396 /714	337 (52.25)	59 (85.51)	85.5	47.8	1.64	0.30	5.39 2.74–10.60	0.000
**Mesenteric retraction**	198/788	174 (24.30)	24 (33.33)	33.3	75.7	1.37	0.88	1.56 0.93–2.61	0.09
**Bowel infiltration**	269/795	222 (30.79)	47 (65.51)	63.5	69.3	2.06	0.53	3.91 2.38–6.42	0.000
**Stomach infiltration**	29/706	25 (3.91)	4 (6.06)	6.1	96.1	1.55	0.98	1.59 0.56–4.32	0.40
**Liver metastasis**	30/795	21 (2.91)	9 (12.33)	12.3	97.1	4.24	0.90	4.69 2.10–10.51	0.000

CI: Confidence Interval; DOR: Diagnostic Odd Ratio; Se: Sensibility; Sp: Specificity; LR -: negative Likelihood Ratio; LR +: positive Likelihood Ratio; CT: Computed Tomography; CA: Carcinome Antigen; PCI: Peritoneal Carcinomatosis Index; LNM: Lymph Node Metastasis.

### 3.3 Multivariate analysis

**Fig. 1:**
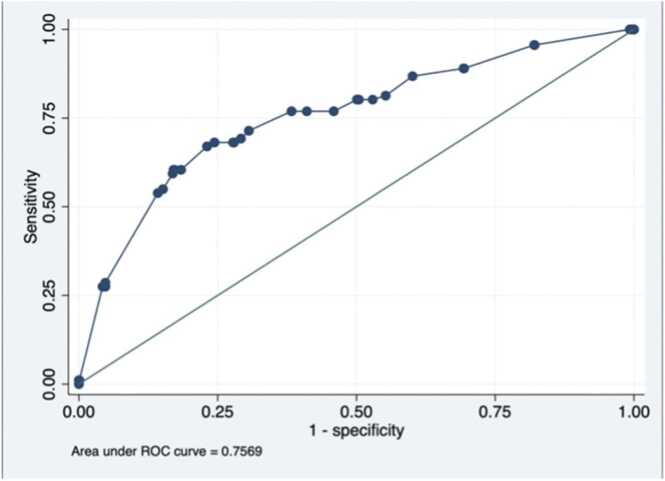
ROC of the prediction model. ROC: Receiving Operating Curve; AUC: Area Under the Curve.

Multivariate analysis is presented in Table 3. The logistic regression model identified four variables independently and statistically associated with splenectomy: age <60 years (aDOR = 1.76 95 %CI [1.13–2.75], p = 0.013), omental cake at initial CT and/or laparoscopy (aDOR = 2.12 95 %CI [1.11–4.08], p = 0.024), diaphragmatic carcinosis at initial CT and/or laparoscopy (aDOR = 2.36 95 %CI [1.34–4.18], p = 0.001) and digestive involvement at initial CT and/or laparoscopy (aDOR = 3.24 95 % CI [1.93–5.43], p < 0.001). All these variables were stable after 1000 bootstrap replications. The ROC-AUC of this prediction model was 0.76 (Fig. 1).

**Table 3:** Prediction logistic regression model and predictive score of splenectomy.

**Variables**	**aDOR [95 % CI]**	**p**	**p after bootstrap (1000 replications)**	**Score** **/10 points**
**Age <60 years**				
No	1			0 point
Yes	1.76[1.13–2.75]	0.013	0.015	2 points
**Omental Cake at initial CT and/or laparoscopy**				
No	1			0 point
Yes	2.12 [1.11–4.08]	0.024	0.018	2 points
**Diaphragmatic carcinosis at initial CT and/or laparoscopy**				
No	1			0 point
Yes	2.36 [1.34–4.18]	0.003	0.001	2 points
**Digestive involvement at initial CT and/or laparoscopy**				
No	1			0 points
Yes	3.24 [1.93–5.43]	<0.001	<0.001	4 points

aDOR: adjusted Diagnostic Odd Ratio; CI: Confidence Interval; CT: Computed Tomography.

**Fig. 2:**
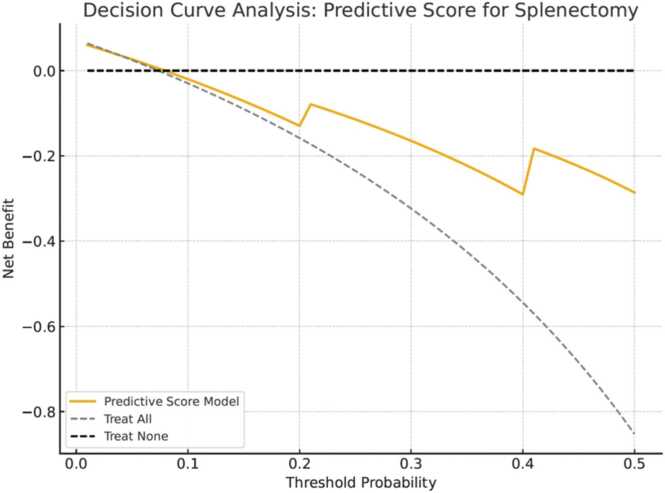
Decision curve analysis of the prediction model.

The predictive score of splenectomy, ranging from 0 to 10 points, was given by the following equation: score = *(age < 60 years × 2) + (Omental Cake at initial CT and/or laparoscopy × 2) + (Diaphragmatic carcinosis at initial CT and/or laparoscopy × 2) + (Digestive involvement at initial CT and/or laparoscopy × 4)*. There was no significant difference between the ROC-AUC of the score and the ROC-AUC of the model (p = 0.07). The high-risk group of splenectomy was defined for a maximal splenectomy score of 10 points. For this threshold value, the probability of splenectomy was 32 % (95 % CI [22.00–44.31]) with a specificity of 95.8 % (95 % CI [94.5–96.9]) and a positive likelihood ratio of 6.31 (95 %CI [4.08–9.78]) (Fig. 2).

The DCA demonstrated that the predictive model provided a positive net clinical benefit compared to the “treat all” and “treat none” strategies within a threshold probability range of 15–40 % (Fig. 2). Within this interval, the model identified patients for whom clinical decision-making based on predicted splenectomy risk would result in better outcomes than uniform management strategies. Outside this range, the net benefit of using the model was equivalent to or lower than the default approaches.

## 4 Discussion

We have developed the first predictive score of splenectomy during primary or interval/final CRS in AEOC. This study included 1 288 patients. Four independent variables were independently and statistically associated with splenectomy: age <60 years (p = 0.015), omental cake at initial CT and/or laparoscopy (p = 0.024), diaphragmatic carcinosis at initial CT and/or laparoscopy (p = 0.001) and digestive involvement at initial CT and/or laparoscopy (p < 0.001). Maximum score of 10 points, patients with the 4 positive criteria, defined the high-risk group of splenectomy. Patients in this group had a 32 % probability of splenectomy with a high specificity of 95.8 % and likelihood ratio positive of 6.31. The DCA showed a positive net clinical benefit of the model between 15 % and 40 % threshold probabilities.

Our study presents some strengths. First, our predictive score of splenectomy is robust since the logistic regression model has been constructed on a very large cohort of 1288 patients from 14 referral centers for ovarian cancer in France within the FRANCOGYN study group. Moreover, we considered overfitting by performing an internal validation of this model with 1000 replications of bootstrap.

Second, the results of our study are in concordance with the literature in terms of splenectomy prevalence in CRS. Indeed, 7 % (n = 91/1 288) of our patients with AEOC underwent splenectomy during primary or interval/final CRS, which is consistent with multiple studies that provide a rate from 1.3 % to 35 % [11], [12], [20], [28]

Finally, our score has a good face validity. As in the literature, our study demonstrated a link between extensive abdominal disease and splenectomy during CRS with three out of four criteria of our score: omental cake at initial CT and/or laparoscopy, diaphragmatic carcinosis at initial CT and/or laparoscopy and digestive involvement at initial CT and/or laparoscopy. Indeed, Said et al., in a retrospective cohort of 3911 patients with CRS in AEOC, reported that splenectomy was very often associated with bowel resection which was not the case if splenectomy was not performed (56.6 % vs 20.8 %, p < 0.001). In the same way, distal pancreatectomy was required in 18.2 % of patients in the splenectomy group vs 0.1 % in the no splenectomy group (p < 0.001). Patients in the splenectomy group also had more elevated CA 125 and tumor grade [12].

Our study also presents some limits. First, the retrospective design of this study could lead to collection bias and missing data. It is also to be noted that a prospective external validation of our score is needed.

Second, we could not study the diagnostic accuracy of CT for splenectomy in our study because that information was not available in the database. We could not either evaluate at all radiological variables of the PET-CT because of too important missing data. The literature showed a poor correlation between prediction of CT alone for splenectomy and notably in the studies of Uzan et al. and Davies et al. with a 20 % prediction rate [18], [20]. Other studies took an interest on CT performance in predicting complete CRS in ovarian cancer but none of them studied splenic invasion specifically. Ferrandina et al. estimated a diagnostic accuracy of CT of 79.2 % for omental extension including spleen, stomach, and lesser sac [29]. Gu et al. reported a CT and PET-CT overall sensitivity of 91 % in recurrent ovarian cancer with no information on splenic extension [30]. In a more recent Indian study, Bagul et al., found a diagnostic accuracy of CT of 86.1 % for omental extension with only 36 patients enrolled and an association between its extension to spleen, colon or stomach with not complete CRS [31].

Third, a specific radiologic assessment of splenic or peri-splenic lesions was not available in the FRANCOGYN database, which was initially designed as a general multicenter registry and not tailored for this specific analysis; therefore, this potentially relevant variable could not be included in our model.

Fourth, while we were able to define a high-risk, “rule-in”, group with strong specificity and LR+, we were unable to identify a threshold meeting “rule-out” criteria (sensitivity > 95 %, LR– < 0.25). This limits the model’s ability to safely exclude the need for splenectomy in low-score patients.

Finally, the FRANCOGYN database did not include information on the specific indications for splenectomy. As such, we were unable to distinguish between splenectomies performed for oncologic reasons, such as capsular, hilar, or parenchymal tumor involvement, and those performed in emergency situations due to intraoperative complications, such as iatrogenic splenic injury during omentectomy or diaphragmatic resection. The database only records whether a splenectomy was performed or not, without detailing the underlying reason. To address this limitation, we attempted to explore the indication for splenectomy by analyzing the postoperative histopathological findings of the spleen. However, among the 91 patients who underwent splenectomy, histologic data were missing for 49 patients (53.8 %), limiting the interpretability of this approach. Among the 42 patients with available data, 16 had histologic confirmation of splenic tumor infiltration, while 26 had no evidence of malignancy. It is important to note that 14 of these 26 patients had undergone neoadjuvant chemotherapy, which could have sterilized the spleen and led to negative histological findings despite initial tumor involvement. Therefore, even with histology, we were not able to definitively differentiate oncologic from iatrogenic splenectomy in our cohort. This limitation, due to the retrospective nature of the database, prevented us from excluding emergency splenectomies or performing a dedicated subgroup analysis. In the literature, 59–73 % of splenectomies are performed for capsular or hilar invasion, 0–36 % for parenchymal spleen metastasis and few for bleeding injury [13], [15], [16], [18], [19], [28].

Splenectomy is a morbid procedure that carries a significant risk of surgical and medical complications. Reported morbidity rates range from 0 % to 30 %, and mortality from 0 % to 8.8 % in some series [28]. Surgical complications include pancreatic fistula, reported in 0–12 % of cases [14], [16], [32], and subphrenic abscesses. Medical complications are mostly infectious or thromboembolic and may lead to intensive care unit admission. Overwhelming post-splenectomy infection has been described in 3–5 % of cases [33], most commonly within the first two years after surgery, although the risk may persist lifelong [34]. These infections are typically caused by encapsulated bacteria (*Streptococcus pneumoniae*, *Neisseria meningitidis*, *Haemophilus influenzae*). In this context, anticipating the need for splenectomy is highly relevant, as it allows for preventive interventions that could reduce associated morbidity and optimize perioperative safety. Our study addresses this need by providing a tool to better identify patients at high risk of requiring splenectomy during cytoreductive surgery.

To ensure a comprehensive evaluation of our model, we used complementary statistical approaches. The ROC curve confirmed good discriminative performance, with an AUC of 0.76, indicating the model’s ability to differentiate patients who will require splenectomy from those who will not. However, statistical discrimination alone does not imply clinical usefulness. Therefore, we performed a DCA, which demonstrated that the model provides a net clinical benefit over “treat all” or “treat none” strategies within a relevant probability range of 15–40 %. This range reflects realistic thresholds where clinicians may reasonably decide to adapt surgical planning. Notably, patients with the maximum score of 10, defined by age <60 years, omental cake, diaphragmatic carcinomatosis, and digestive involvement, fall within this optimal range, with an observed splenectomy probability of 32 % and a specificity of 95.8 %. This reinforces the clinical applicability of the score, especially in identifying a well-defined high-risk group.

For these high-risk patients, the model supports personalized perioperative planning. Anticipated measures may include preoperative vaccination against encapsulated bacteria, patient education regarding infection risk and antibiotic prophylaxis, and the planned presence of a visceral or upper gastrointestinal surgeon during cytoreductive surgery. These actions are particularly important in cases where splenectomy is not expected preoperatively but may become necessary intraoperatively, including in unplanned scenarios. Importantly, the model was developed using a large, real-world multicenter cohort that included both planned and unplanned splenectomies, ensuring its applicability to the full spectrum of clinical practice. Moreover, identifying a high predicted risk of splenectomy before chemotherapy may help guide the initial treatment strategy. As already practiced in the context of extensive bowel disease, selecting neoadjuvant chemotherapy could potentially reduce tumor burden, facilitate resection, and avoid major procedures such as splenectomy without compromising oncologic outcomes. Thus, our model not only provides a robust tool for surgical risk stratification but also promotes more individualized, safer, and better-informed care.

## CRediT authorship contribution statement

**Lavoue Vincent:** Conceptualization. **Mimoun Camille:** Writing – review & editing, Writing – original draft, Supervision, Methodology, Formal analysis, Data curation, Conceptualization. **Canlorbe Geoffroy:** Conceptualization. **Kerbage Yohan:** Conceptualization. **Akladios Cherif:** Conceptualization. **Ouldamer Lobna:** Conceptualization. **Costaz Helene:** Conceptualization. **Bricou Alexandre:** Conceptualization. **Do Thien-Kim:** Writing – review & editing, Writing – original draft, Supervision, Methodology, Formal analysis, Data curation, Conceptualization. **Azais Henri:** Conceptualization. **Dabi Yohann:** Writing – review & editing, Writing – original draft, Supervision, Methodology, Formal analysis, Data curation, Conceptualization. **Chauvet Pauline:** Conceptualization. **Teboul Cyril:** Conceptualization. **Carcopino Xavier:** Conceptualization. **Uzan Jennifer:** Conceptualization. **Huchon Cyrille:** Conceptualization. **Margueritte Francois:** Conceptualization.

## Funding sources

This research did not receive any specific grant from funding agencies in the public, commercial, or not-for-profit sectors

## Declaration of Competing Interest

The authors declare that they have no known competing financial interests or personal relationships that could have appeared to influence the work reported in this paper.
